# Machine learning inversion from scattering for mechanically driven polymers

**DOI:** 10.1107/S160057672500634X

**Published:** 2025-08-28

**Authors:** Lijie Ding, Chi-Huan Tung, Bobby G. Sumpter, Wei-Ren Chen, Changwoo Do

**Affiliations:** a Neutron Scattering Division Oak Ridge National Laboratory,Oak Ridge TN 37831 USA; b Center for Nanophase Materials Sciences Oak Ridge National Laboratory,Oak Ridge TN 37831 USA; Tohoku University, Japan

**Keywords:** small-angle scattering, machine learning, Gaussian process regressors, Monte Carlo methods, polymers

## Abstract

A machine learning inversion method is developed for analyzing scattering functions of mechanically driven polymers and extracting the corresponding feature parameters, which include energy parameters and conformation variables. The polymer is modeled as a chain of fixed-length bonds constrained by bending energy, and it is subject to external forces such as stretching and shear. We generate a data set consisting of random combinations of energy parameters, including bending modulus, stretching and shear force, along with Monte Carlo-calculated scattering functions and conformation variables such as end-to-end distance, radius of gyration and off-diagonal component of the gyration tensor. The effects of the energy parameters on the polymer are captured by the scattering function, and principal component analysis ensures the feasibility of the machine learning inversion. Finally, we train a Gaussian process regressor using part of the data set as a training set and validate the trained regressor for inversion using the rest of the data. The regressor successfully extracts the feature parameters.

## Abstract 2

It is demonstrated that, when a semiflexible polymer is under external forces, these forces and the bending modulus, along with conformation variables of the polymer, can be extracted from the scattering function using a machine learning inversion method.

## 1. Introduction

Machine learning (ML) (Murphy, 2012 ▸; Carleo *et al.*, 2019 ▸) has emerged as a powerful tool for data analysis, enabling the extraction of patterns, trends and insights from large complex data sets. Its ability to automate the discovery of meaningful relationships within data has helped to advance numerous fields, including scattering analysis (Chang *et al.*, 2022 ▸). ML techniques can be used for the rapid interpretation of underlying material properties and structural parameters according to complex scattering data. This technique has been applied to various systems including colloids (Chang *et al.*, 2022 ▸; Huang *et al.*, 2023 ▸; Tung *et al.*, 2023 ▸), polymers (Tung *et al.*, 2022 ▸; Ding *et al.*, 2025*a* ▸) and lyotropic lamellar systems (Tung *et al.*, 2024*b* ▸).

Polymers are ubiquitous in nature and play a pivotal role in everyday life and in numerous industry settings (De Gennes, 1979 ▸; De Gennes, 1990 ▸; Sperling, 2015 ▸). Understanding the physics of polymers can help us to better design and engineer new materials for different applications. The polymers’ response to external forces is often of interest as the mechanical properties of the polymer can thus be revealed (Wang & Wang, 2011 ▸; Li & Wang, 2016 ▸; Smith *et al.*, 1999 ▸; Schroeder *et al.*, 2005 ▸). Due to the small physical size of most polymers, scattering experiments (Murphy *et al.*, 2020 ▸), such as X-ray (Chu & Hsiao, 2001 ▸) or neutron (Shibayama, 2011 ▸; Chen, 1986 ▸) scattering, are commonly employed to probe their structure and dynamics at the molecular level. The scattering function measured by these experiments provides indirect but valuable information about the polymer’s conformation and behavior under mechanical stress. Recent advancements in RheoSANS (rheological small-angle neutron scattering; Murphy *et al.*, 2020 ▸) and sample environments have enabled the application of external forces that are comparable to the bending energy of the polymer using flow cells. A Monte Carlo (MC) (Krauth, 2006 ▸) method that we recently developed (Ding *et al.*, 2024 ▸) has enabled the theoretical study of mechanically driven polymers, which are semiflexible polymers under external forces including stretching and shear, and the calculation of scattering functions compatible with scattering experiments. Unlike the one-dimensional scattering profile found in isotropic systems, the scattering function of a mechanically driven polymer is not isotropic due to the existence of non-isotropic forces (Huang *et al.*, 2025 ▸), rendering the existing models including Gaussian chain (Debye, 1947 ▸), worm-like chain (Kholodenko, 1993 ▸) and flexible cylinder (Pedersen & Schurtenberger, 1996 ▸) unsuitable for scattering analysis.

The lack of a scattering analysis technique prevents us from extracting physical parameters at the molecular level from mechanically driven polymers using small-angle scattering experiments (Huang *et al.*, 2025 ▸). For this we turn instead to ML for a practical solution. ML techniques like Gaussian process regression (GPR) (Williams & Rasmussen, 2006 ▸) have been used to extract physical parameters directly from scattering data (Chang *et al.*, 2022 ▸). Generative models utilizing a variational autoencoder (Tung *et al.*, 2024*a* ▸; Ding *et al.*, 2025*b* ▸) have been combined with a regression model to fit the scattering function. The GPR method is more straightforward yet has only been applied to 1D scattering data so far, while the scattering data for a mechanically driven polymer are naturally 2D due to the anisotropy introduced by the external forces. In this paper, we extend the GPR to 2D scattering and apply the ML inversion technique (Chang *et al.*, 2022 ▸) to map the scattering function onto inversion targets or feature parameters of the mechanically driven polymers. We generate a data set for training and testing using the MC simulation that we previously developed. The effects of energy parameters such as bending, stretching and shear on the scattering function are well reflected independently and the corresponding polymer deformation is well captured by the calculated scattering function. The feasibility of the proposed ML inversion framework is validated by principal component analysis, which also provides the characteristic orientation of the scattering function as a byproduct. Excellent agreement between the ML-extracted feature parameters and the MC reference values is achieved, showing good accuracy for our approach.

## 2. Method

We model the polymer as a chain of *N* connected bonds with fixed length *l*_b_. The tangent of bond *i* is **t**_*i*_ ≡ (**r**_*i*+1_ − **r**_*i*_)/*l*_b_, where **r**_*i*_ is the position of the joint connecting bonds *i* − 1 and *i*. We fix one end of the polymer at the origin. The polymer energy is given by 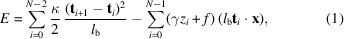
where κ is the bending modulus, *f* is the stretching force applied in the *x* direction, γ is the shear ratio along the *z* direction, *z*_*i*_ = **r**_*i*_ · **z** is the *z* component of the position of joint *i* and (**t**_*i*_ · **x**) is the *x* component of the bond tangent **t**_*i*_. A hard-sphere interaction between polymer joints, with a sphere radius *l*_b_/2, was used to account for self-avoidance of the polymer.

We sample the configuration space of the polymer using MC and calculate the scattering function and conformation variables of the polymer. We then use GPR to achieve a mapping from the scattering function to the system parameters and conformation variables.

### 2.1. Monte Carlo simulation

We simulate the polymer under different system parameters using the Markov chain MC method (Ding *et al.*, 2024 ▸) that we previously developed, where each configuration of the polymer is generated by updating the previous one. Two types of non-local moves are used for updating the polymer: crankshaft and pivot. Crankshaft executes a random rotation of an inner sub-chain of the polymer, while pivot rotates the sub-chain including the end. Details of the simulations can be found in our previous work (Ding *et al.*, 2024 ▸). From the simulations, the scattering function and other conformation variables, including end-to-end distance, radius of gyration and off-diagonal component of the gyration tensor, were computed. The scattering function is defined as (Chen, 1986 ▸) 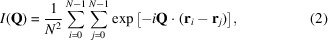
where **Q** is the scattering vector [*Q* = (4π/λ) sin(θ/2), where θ is the scattering angle and λ is the wavelength of the incident radiation] and *N* is the total number of segments. In practice, a projection of *I*(**Q**) onto a specific plane is collected in scattering experiments. Since the force field is applied in the (*x*, *z*) plane or flow-velocity gradient plane, we calculate the two-dimensional *I*_*xz*_(**Q**) = *I*(*Q*_*x*_, *Q*_*y*_ = 0, *Q*_*z*_) accordingly. In addition, the end-to-end distance is defined as *R*^2^ = |**r**_0_ − **r**_*N*−1_|^2^, the radius of gyration is 
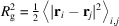
 and the *xz* component of the gyration tensor is 
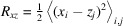
, with 〈…〉_*i*, *j*_ denoting the average over all pairs of joints.

### 2.2. Gaussian process regression

To obtain a mapping from the scattering function **x** = *I*_*xz*_(**Q**) to inversion targets **y** including both system parameters and conformation variables, we train a GPR (Williams & Rasmussen, 2006 ▸) by feeding training data 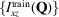 containing scattering functions calculated with various system parameters (κ, *f*, γ). We define the prior on the regression function as a Gaussian process *g*(**x**) ≃ *GP*[*m*(**x**), *k*(**x**, **x**′)], where *m*(**x**) is the prior mean function and *k*(**x**, **x**′) is the covariance function or kernel. Given a test data set 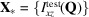, the goal of the regressor is to estimate 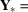
*g*(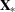). The joint distribution for a Gaussian process is 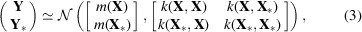
where we use a constant prior mean *m*(**x**) and a linear combination of a radial basis function (Gaussian) kernel and white noise for the kernel *k*(**x**, **x**′) = 
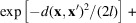

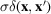
. Here, *d*(**x**, **x**′) = |**x** − **x**′| is the Euclidean distance between **x** and **x**′, *l* is the correlation length, σ is the variance of observational noise, and δ is the Kronecker delta function. *l* and σ are the hyperparameters for the regression and can be obtained by training.

## 3. Results

We prepare the training {*I*_train_(**Q**)} and test {*I*_test_(**Q**)} sets by carrying out MC simulations of the polymer chains with various combinations of energy parameters: bending modulus κ, stretching force *f* and shear rate γ. The scattering function and conformation variables were measured for each simulation. We use natural units in our simulation such that lengths are measured in units of the bond length *l*_b_ and energies are measured in units of thermal noise *k*_B_*T*. Prior to training, we first study the effect of energy parameters on the scattering function and then validate the feasibility of inversion using principal component analysis. Finally, we train our GPR and compare the ML-calculated inversion targets with values calculated using MC.

### 3.1. Scattering function of the polymers

**Figure 1:**
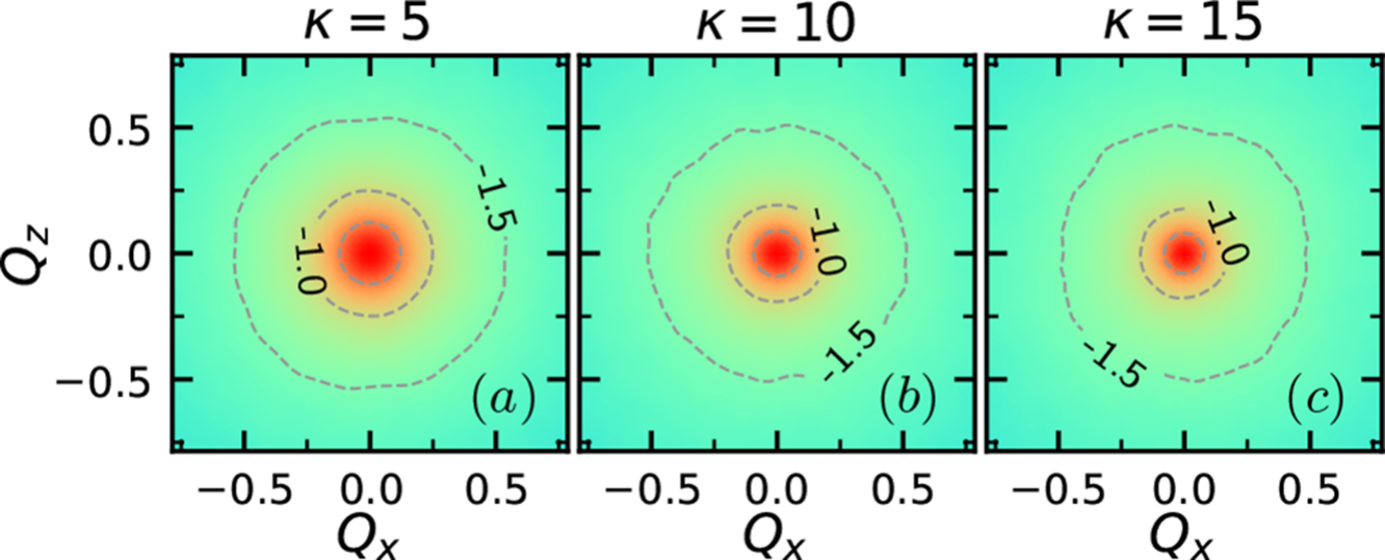
*I*_*xz*_(**Q**) of a semiflexible chain with *L* = 200 in its quiescent state with bending modulus κ = 5, 10 and 15.

In order for the GPR to achieve mapping from the scattering function to the inversion targets, the scattering function must reflect the changes in the inversion targets, *i.e.* the energy parameters. These results are demonstrated in Figs. 1 and 3, where the scattering function at various bending modulus κ, stretching force *f* and shear rate γ are shown.

The bending modulus κ determines the persistence length of the polymer. A longer persistence length makes the polymer more rod-like, thus lowering the scattering intensity *I*_*xz*_(**Q**) at larger *Q* = |**Q**|. Fig. 1 ▸ shows *I*_*xz*_(**Q**) at different κ; the contour of *I*_*xz*_(**Q**) shows circular symmetry, indicating isotropy of the polymer system in the absence of external forces. The ring of contour level also shrinks as the bending modulus κ increases from Fig. 1 ▸(*a*) to Fig. 1 ▸(*c*), consistent with our intuition about the effect of κ on the persistence length.

**Figure 2:**
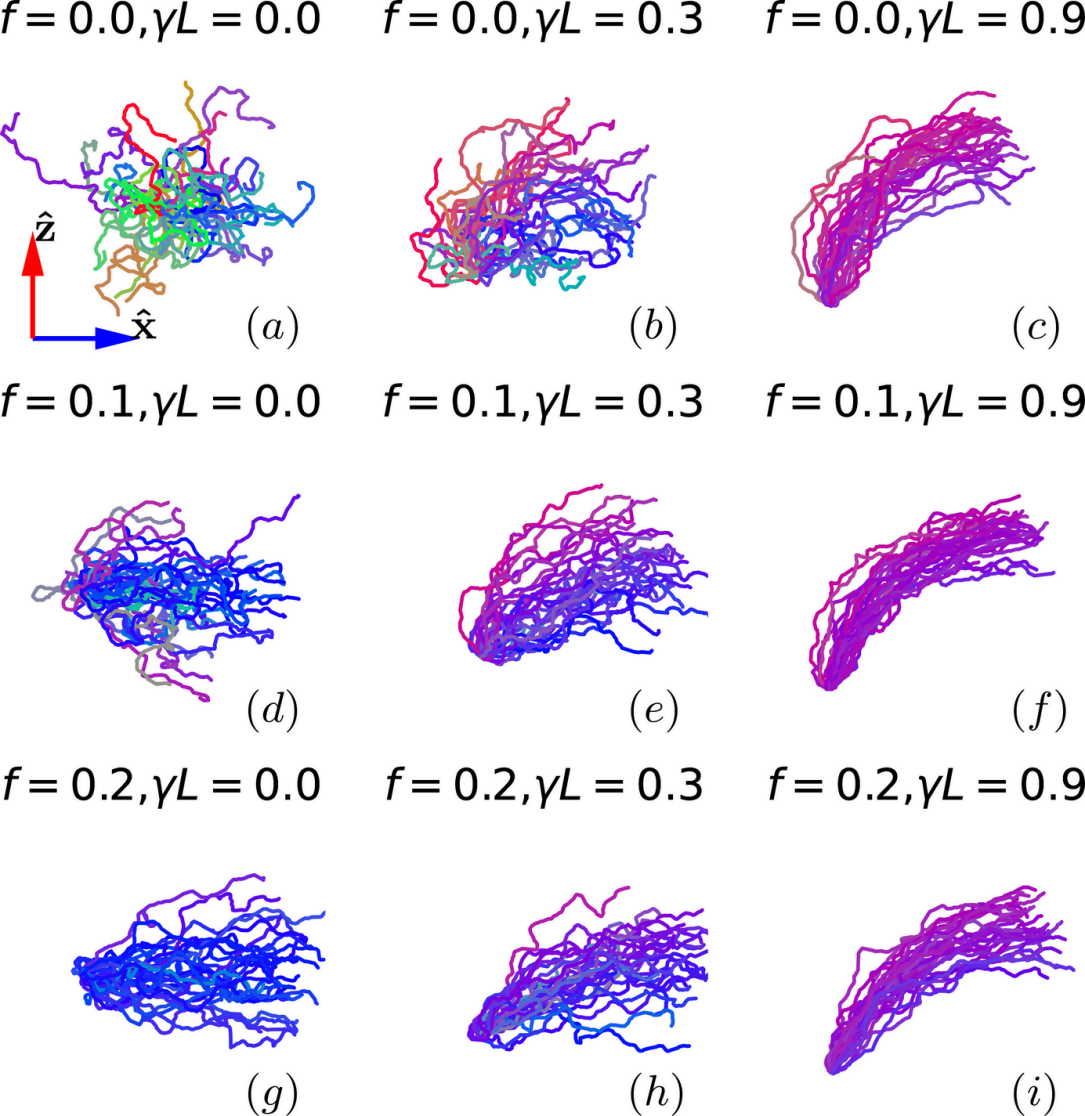
Sample configurations of a semiflexible chain with *L* = 200 and κ = 10 with various combinations of stretching and shear (*f*, γ) = (0, 0.1, 0.2) × (0, 0.3, 0.9). The color corresponds to the end-to-end orientation in the *xz* plane. The system is symmetric about ±*xz* for panels (*b*) and (*c*), where *f* = 0, γ ≠ 0; these configurations are flipped to the *xz* direction for better visualization.

When external forces are applied, the polymer deforms accordingly. Fig. 2 ▸ shows sample configurations of the polymer under different stretching *f* and shear γ. When stretching *f* is only applied along the *x* direction, the polymer extends along the *x* direction as shown in Figs. 2 ▸(*d*) and 2 ▸(*g*). Figs. 2 ▸(*b*) and 2 ▸(*c*) show that the polymer extends towards the *xz* direction in a convex manner when only the shear γ is applied. Combining the stretching force and shear rate, the polymer behaves like something in the middle, such that an increasing stretching force *f* pulls the polymer more towards the *x* direction [compare Figs. 2 ▸(*b*), 2 ▸(*e*) and 2 ▸(*f*)]. These deformations are also reflected in the scattering function. The anisotropic behavior of a polymer should deform the circular symmetric shape of *I*_*xz*_(**Q**).

**Figure 3:**
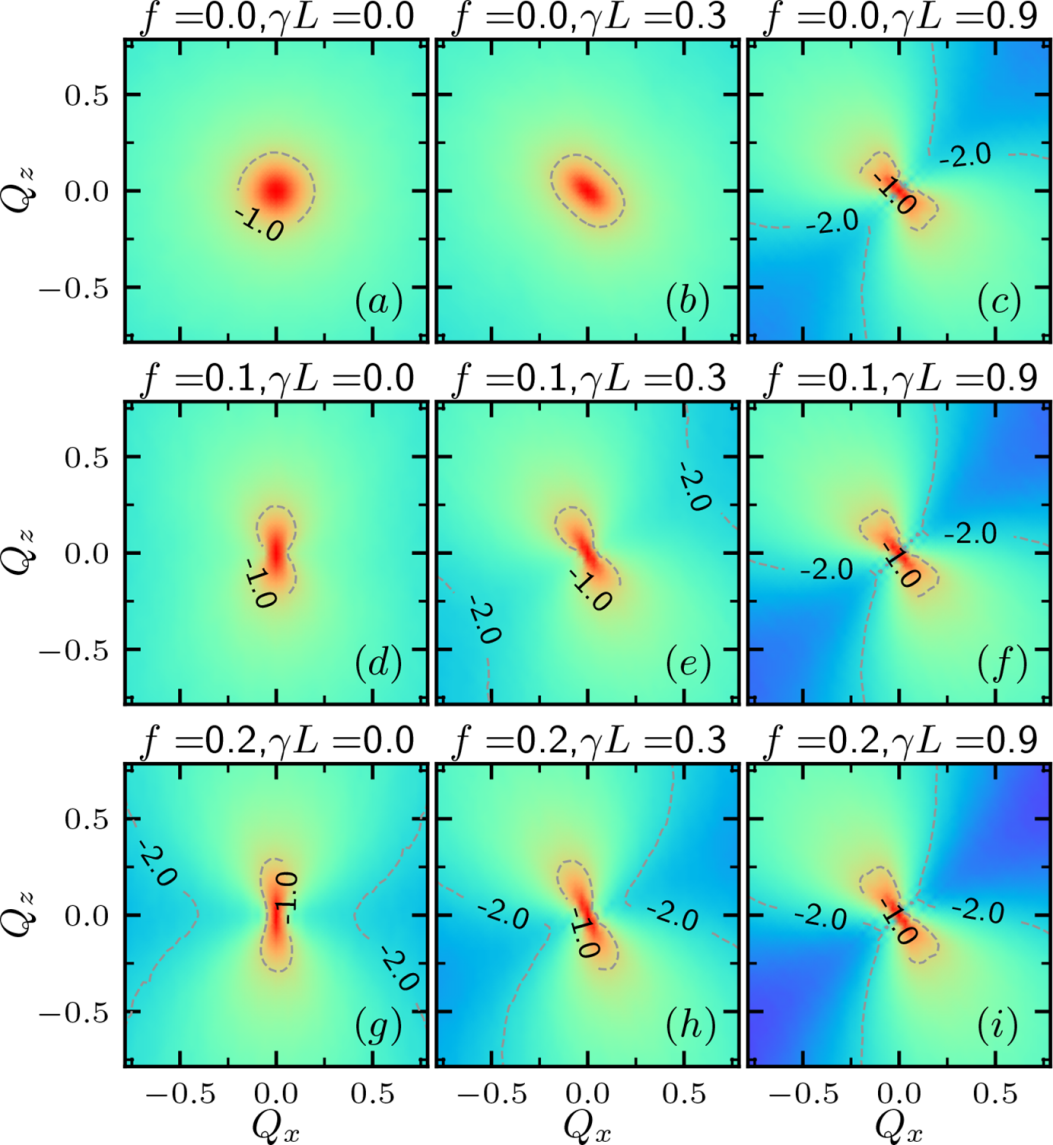
Scattering function *I*_*xz*_(**Q**) of a semiflexible chain with *L* = 200 and κ = 10 with various combinations of stretching and shear (*f*, γ) = (0, 0.1, 0.2) × (0, 0.3, 0.9).

Fig. 3 ▸ shows the scattering function *I*_*xz*_(**Q**) for the polymers corresponding to Fig. 2 ▸. The contour of the scattering function evolves into an oval and then a dumbbell shape as the applied force increases. *I*_*xz*_(**Q**) at high *Q* decreases with the increasing magnitude of stretching *f* and shear γ, reflecting an increase in the radius of gyration due to straightening. On the other hand, the ratio between *f* and γ affects the orientation of the *I*_*xz*_(**Q**) contour. For pure stretching, the contour of *I*_*xz*_(**Q**) extends along the *z* direction, indicating elongation of the polymer along the *x* direction. In contrast, pure shear makes the contour of *I*_*xz*_(**Q**) extend along the −*xz* direction, reflecting the elongation of the polymer along the *xz* direction. Applying and increasing the shear rate on a polymer under stretching, as shown in Figs. 3 ▸(*g*), 3 ▸(*h*) and 3 ▸(*i*), rotates the orientation of the dumbbell-shaped contour towards the −*xz* direction.

### 3.2. Feasibility of ML inversion

Due to the significant difference of the effect on scattering functions induced by different energy parameters, we anticipate that the difference in energy parameters can be distinguished from the scattering function using GPR. To validate the feasibility of such inversion numerically, we generate 1680 random combinations of (κ, *f*, γ), in which κ ≃ *U*(2, 20), *f* ≃ *U*(0, 0.5), γ*L* ≃ *U*(0, 2), where *U*(*a*, *b*) is the uniform distribution in the interval [*a*, *b*]. We then run MC simulations to calculate the scattering function *I*_*xz*_(**Q**) of the polymer system at these energy parameters. Each *I*_*xz*_(**Q**) is calculated for 2601 = 51 × 51 different (*Q*_*x*_, *Q*_*z*_), where *Q*_*x*_, *Q*_*z*_ ∈ [−50π/*L*, 50π/*L*], uniformly placed on the 51 × 51 grid. These *I*_*xz*_(**Q**) are then flattened to 2601-dimensional vectors and arranged into a 1680 × 2601 matrix **F**. Following a similar ML inversion framework (Chang *et al.*, 2022 ▸), **F** is then decomposed into **F** = **UΣV**^T^ using singular value decomposition (Strang, 2022 ▸), such that **U** is 1680 × 1680, **Σ** is 1680 × 2601 and **V** is 2601 × 2601. The singular value matrix **Σ**^2^ is diagonal and its entries are proportional to the variance of the data set **F** projected onto the corresponding principal axis (Zhu & Ghodsi, 2006 ▸), which is given by the singular vector **V**.

**Figure 4:**
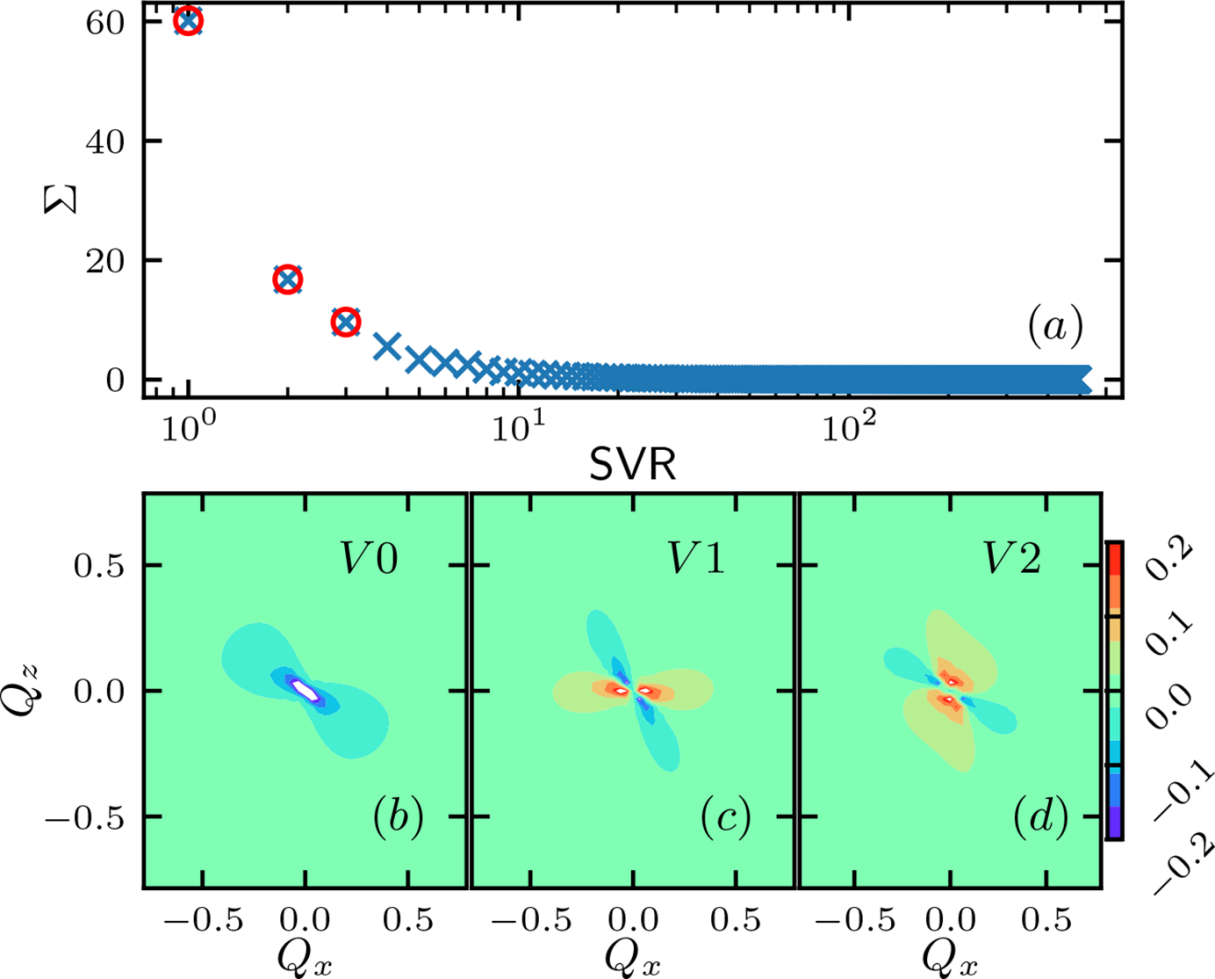
Singular value decomposition of the scattering function data set. (*a*) Singular value Σ versus singular value rank. Values with top-three rank are highlighted with red circles. (*b*)–(*d*) First three singular vectors.

Fig. 4 ▸(*a*) shows the diagonal entry value of **Σ** versus its sin­gular value rank (SVR). As the SVR increases, its corresponding value decreases rapidly, indicating that the variations in *I*_*xz*_(**Q**) are dominated by the first few singular vectors of lower rank. Figs. 4 ▸(*b*)–4 ▸(*d*) show the first three single vectors, which give a characteristic basis for *I*_*xz*_(**Q**).

**Figure 5:**
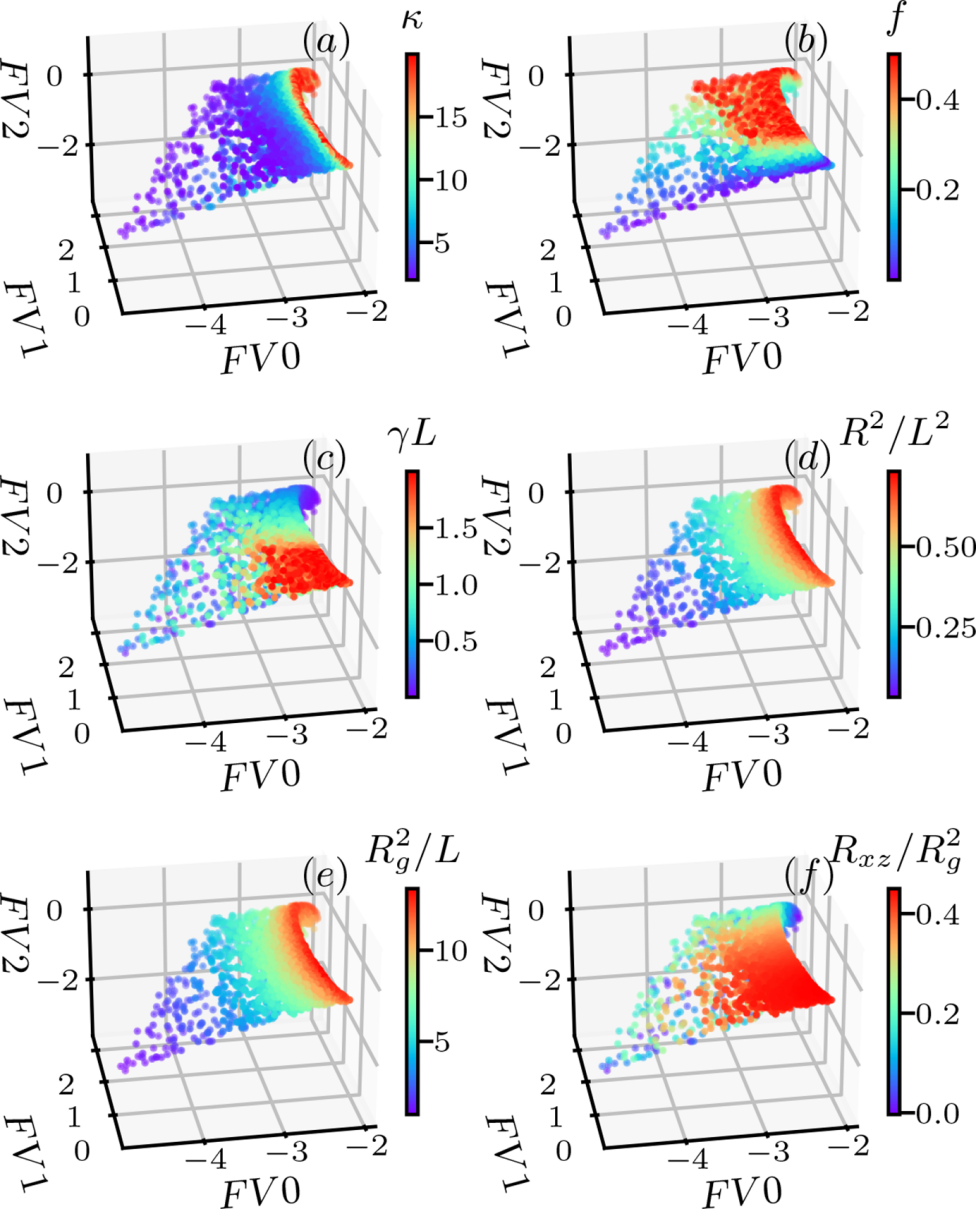
Distribution of various inversion features of the training data in the singular value space. (*a*) Bending modulus κ. (*b*) Stretching force *f*. (*c*) Contour length normalized shear γ*L*. (*d*) End-to-end distance scaled by contour length squared *R*^2^/*L*^2^. (*e*) Radius of gyration squared scaled by contour length 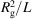. (*f*) Off-diagonal *xz* component of gyration tensor 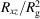.

By projecting the data set **F** onto the first three singular vectors *V*0, *V*1 and *V*2, the (*FV*0, *FV*1, *FV*2) coordinates provide a good proxy of **F** = {*I*_*xz*_(**Q**)}. Fig. 5 ▸ shows the distribution of the six inversion targets in the (*FV*0, *FV*1, *FV*2) space. Three of these are the energy parameters – bending modulus κ, stretching force *f* and shear rate γ – and the other three are conformation variables – end-to-end distance *R*^2^, radius of gyration 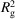 and off-diagonal *xz* component of the gyration tensor *R*_*xz*_. In this (*FV*0, *FV*1, *FV*2) space, each point corresponds to one *I*_*xz*_(**Q**) in **F**, and the color represents the corresponding value of the inversion target. From the color distribution, we note that the inversion targets, feature variables, are all well spread out in (*FV*0, *FV*1, *FV*2) space, indicating that a smooth and continuous mapping between *I*_*xz*_(**Q**) and the inversion target can be obtained, thus validating the feasibility of the ML inversion.

### 3.3. ML inversion of feature variables

To illustrate the inversion of feature parameters 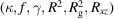 from scattering functions *I*_*xz*_(**Q**), we partition the total data set **F** = {*I*_*xz*_(**Q**)} into two sets: a training set 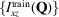 consisting of 70% of **F**, and a test set 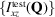 consisting of the other 30% of **F**. We use the training set to obtain the optimized hyperparameters (*l*, σ) through gradient descent on the log marginal likelihood landscape. We do this for the kernel for each feature parameter individually, and then use the trained GPR with the optimized (*l*, σ) to predict the feature parameters of the test set from their *I*_*xz*_(**Q**). The *scikit-learn* Gaussian process library (Pedregosa *et al.*, 2011 ▸) was used for the training and inversion.

**Figure 6:**
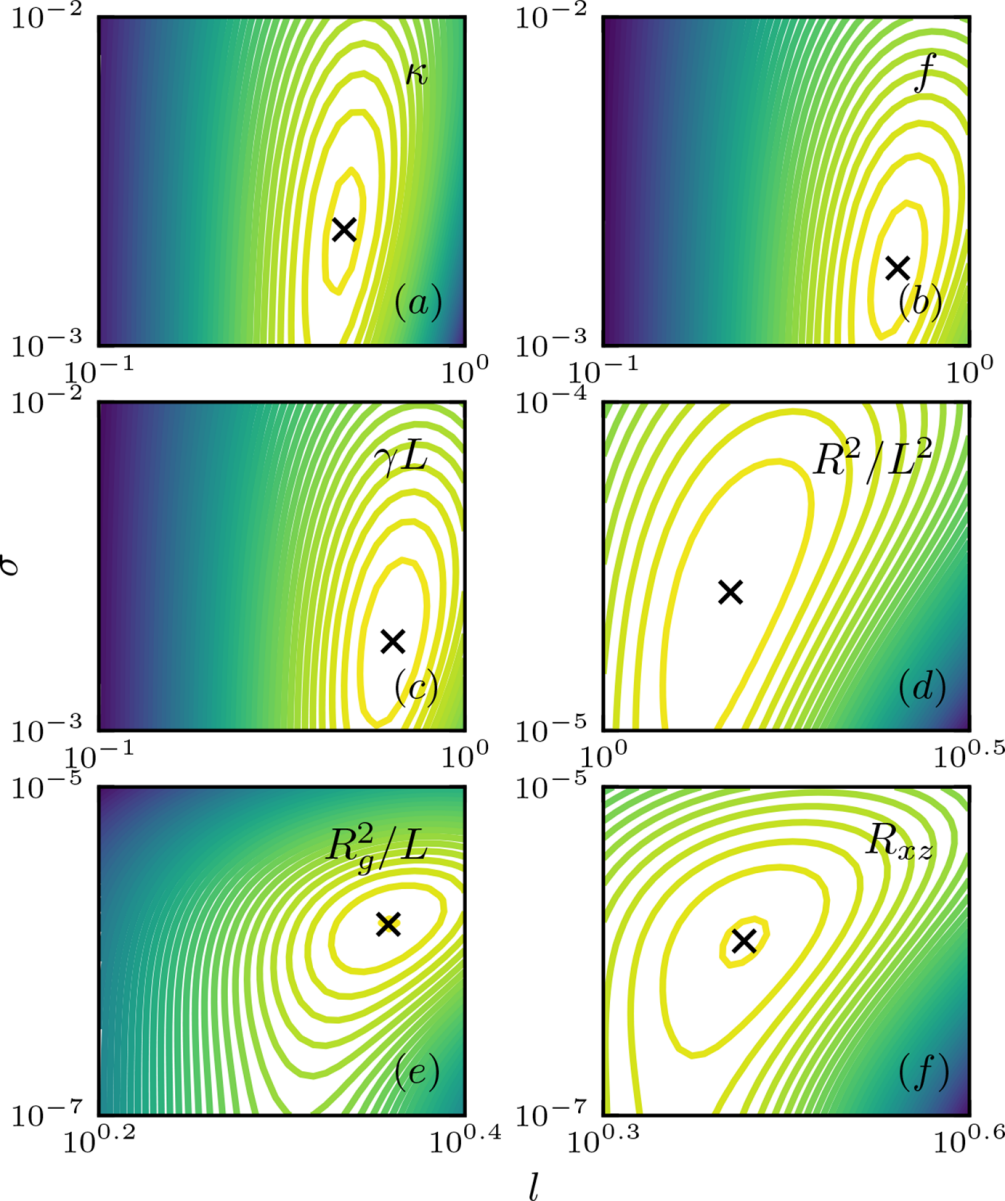
Log marginal likelihood surface of hyperparameters *l* and σ for various inversion features, with optimized values marked with black crosses. (*a*) Bending modulus κ. (*b*) Stretching force *f*. (*c*) Contour length normalized shear γ*L*. (*d*) End-to-end distance scaled by contour length squared *R*^2^/*L*^2^. (*e*) Radius of gyration squared scaled by contour length 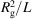. (*f*) Off-diagonal *xz* component of gyration tensor 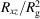.

The log marginal likelihood of the prior is used as the cost function for optimizing the hyperparameters (*l*, σ) (Williams & Rasmussen, 2006 ▸). Fig. 6 ▸ shows the log marginal likelihood contour in (*l*, σ) space for each feature parameter or inversion target. The optimized (*l*, σ) are obtained by gradient descent and shown in Table 1 ▸. The contours in Fig. 6 ▸ show unimodal convex patterns, which further suggest the reliability of the trained hyperparameters. While the optimized hyperparameters (*l*, σ) differ for each inversion target, two scales of correlation length and noise level emerge. The optimized *l* and σ for all the energy parameters have the same order of magnitude, which is also true for the conformation parameters but with a higher order of magnitude, indicating that the scattering function is more sensitive to the variation in energy parameters compared with conformation change.

**Figure 7:**
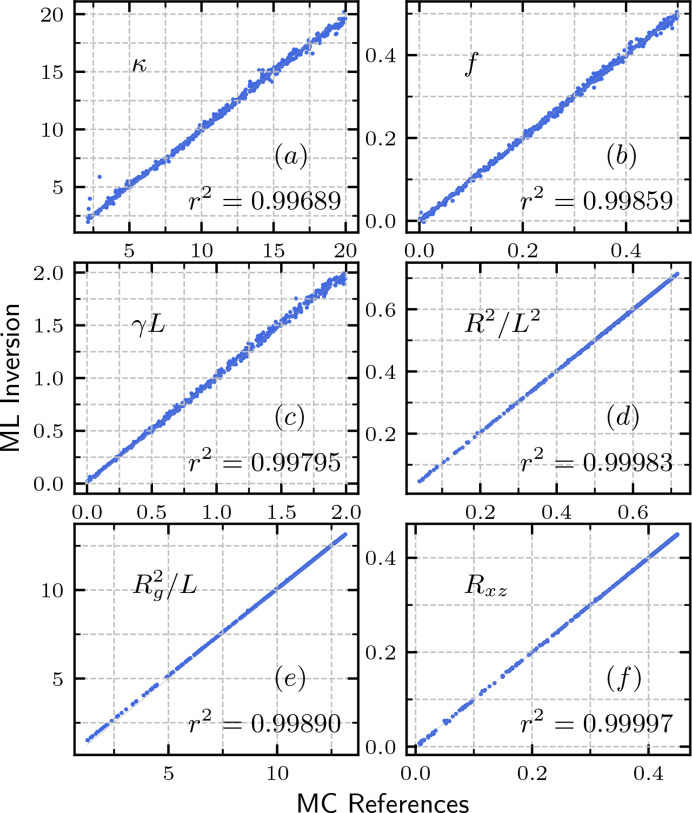
Comparison between the feature variables extracted from the scattering function *I*(**Q**) using GPR and their corresponding computational references calculated using MC simulations. Coefficient of determination, *r*^2^ scores, are indicated at the bottom of each panel.

Finally, we use the scattering function from the test set 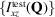 as input to the trained GPR and calculate the feature parameters 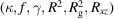 as ML inversion from *I*_*xz*_(**Q**). Fig. 7 ▸ shows a comparison between the GPR-predicted feature parameters and the MC references. All of the data lie close to the diagonal line, with an *r*^2^ score, coefficient of determination, close to 1. The high precision of the inversion shows the power of our ML approach for extracting important system information from the scattering function.

## 4. Summary

In summary, we have applied an ML inversion method to extract feature parameters from the scattering data of mechanically driven polymers. The ML inversion framework was trained on a theoretically calculated data set of a polymer system that is determined by several energy parameters: bending modulus κ, stretching force *f* and shear rate γ. The inversion targets included these energy parameters and also conformation variables such as the end-to-end distance *R*^2^, the radius of gyration 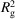 and the off-diagonal component of the gyration tensor *R*_*xz*_. The scattering function *I*_*xz*_(**Q**) of the polymer under different energy parameters was calculated using an MC method that we developed previously (Ding *et al.*, 2024 ▸). We have demonstrated the feasibility of the ML inversion by carrying out principal component analysis of the data set **F** = {*I*_*xz*_(**Q**)} and investigated the distribution of feature parameters by projecting the data set **F** to a three-dimensional singular vector space. The GPR was trained and validated, showing that inversion of the feature parameters can be achieved with high precision.

The versatility of our method promotes its application to the inversion analysis of polymer systems characterized by different intrinsic interactions or under other external forces. For instance, the polymer chains are often charged, in which case instead of using a single parameter, the bending modulus, the interaction between monomers on the polymer can be modeled by the two-parameter Yukawa interaction (Robbins *et al.*, 1988 ▸). The sample environment of the RheoSANS experiments can introduce nonuniform shear flow like Hagen–Poiseuille flow (Batchelor, 2000 ▸). More complicated polymer systems, including polymer brushes (Feng & Huang, 2018 ▸), star polymers (Ren *et al.*, 2016 ▸) and polymer melts (Kremer & Grest, 1990 ▸), are also of interest. Modification of the MC simulation can be made accordingly and ML inversion analysis similar to this work can be carried out.

While our approach is general, inversion from scattering depends on the specific physical model. Just like traditional analysis with model functions relies on how rigorously the model functions were derived and are capable of representing the actual scattering data, our ML approach also relies on the accuracy of the simulated training data for representing the actual experimental system. If the experimental system differs significantly in these physical characteristics from our simulation, the inversion model would need to be retrained on more appropiate simulation data.

We note that the inversion method requires the input scattering function to have the same **Q** grid as the training set, which can lead to interpolation of the experimental data in practice. Recent developments in ML (Liu *et al.*, 2024 ▸; Tung *et al.*, 2025 ▸) show the feasibility of mapping from vectors to functions, which opens the possibility of an alternative method of scattering analysis. Instead of training the mapping from scattering data in discrete **Q** to feature parameters as an inversion, the new framework can learn the mapping from energy parameters to the scattering function in continuous **Q** values, which enables calculation of the scattering function that can then be used for a quick gradient descent optimization of the energy parameter directly. This approach can also be used to cross validate our inversion method. Finally, more detailed analysis of the performance of our method can be carried out, for example studying the behavior of the model on different sized grids of the scattering image and with different splits of the training and testing data.

## Figures and Tables

**Table 1:** Optimized hyperparameters for each feature, obtained from maximum log marginal likelihood

	*l*	σ
κ	4.6828 × 10^−1^	2.2548 × 10^−3^
*f*	6.3714 × 10^−1^	1.7219 × 10^−3^
γ*L*	6.3591 × 10^−1^	1.8671 × 10^−3^
*R*^2^/*L*^2^	1.4921	2.6388 × 10^−5^
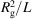	2.2814	1.4582 × 10^−6^
*R*_*xz*_	2.6027	1.1527 × 10^−6^
